# Priming and reversals of the perceived ambiguous orientation of a structure-from-motion shape and relation to personality traits

**DOI:** 10.1371/journal.pone.0273772

**Published:** 2022-08-26

**Authors:** Leo Poom, Melina Matin

**Affiliations:** Department of Psychology, Uppsala University, Uppsala, Sweden; Justus Liebig Universitat Giessen, GERMANY

## Abstract

We demonstrate contributions of top-down and bottom-up influences in perception as explored by priming and counts of perceived reversals and mixed percepts, as probed by an ambiguously slanted structure-from-motion (SFM) test-cylinder. We included three different disambiguated primes: a SFM cylinder, a still image of a cylinder, and an imagined cylinder. In Experiment 1 where the prime and test sequentially occupied the same location, we also administered questionnaires with the Big-5 trait openness and vividness of visual imagery to probe possible relations to top-down driven priming. Since influences of gaze or position in the prime conditions in Experiment 1 could not be ruled out completely, Experiment 2 was conducted where the test cylinder appeared at a randomly chosen position after the prime. In Experiment 2 we also measured the number of perceptual reversals and mixed percepts during prolonged viewing of our ambiguous SFM-cylinder, and administered questionnaires to measure all Big-5 traits, autism, spatial and object imagery, and rational or experiential cognitive styles, associated with bottom-up and top-down processes. The results revealed contributions of position-invariant and cue-invariant priming. In addition, residual contributions of low-level priming was found when the prime and test were both defined by SFM, and were presented at the same location, and the correlation between the SFM priming and the other two priming conditions were weaker than between the pictorial and imagery priming. As previously found with ambiguous binocular rivalry stimuli, we found positive correlations between mixed percepts and the Big-5 dimension openness to experience, and between reversals, mixed percepts and neuroticism. Surprisingly, no correlations between the scores from the vividness of imagery questionnaires and influence from any of the primes were obtained. An intriguing finding was the significant differences between the positive correlation from the experiential cognitive style scores, and the negative correlation between rational style and the cue invariant priming. Among other results, negative correlations between agreeableness and all priming conditions were obtained. These results not only support the notion of multiple processes involved in the perception of ambiguous SFM, but also link these processes in perception to specific personality traits.

## Introduction

Ambiguous perception occurs when the same stimulus gives rise to multiple interpretations that switch over time and is an intriguing perceptual phenomenon that has occupied scientists for centuries. Here, we probed the relative contributions of bottom-up and top-down processes in the perception of an ambiguous structure-from-motion (SFM) test-stimulus consisting of a slanted cylinder with an ambiguous 3D orientation, rather than an ambiguous rotation-direction as typically used in studies using ambiguous SFM stimuli. By constructing cylinder stimuli with ambiguous orientation instead of ambiguous rotation-direction allows us to create disambiguated prime cylinders using other cues than motion to define the prime, for example pictorial cues, which is not possible for ambiguous rotation. In two experiments, we investigated individuals’ susceptibility to priming, where a prime-cylinder with disambiguated slant influences the perceived slant of the subsequently presented ambiguous test-cylinder. In Experiment 2, we also let participants count the frequency of perceived reversals and mixed percepts during prolonged viewing of the ambiguously slanted test-cylinder. High-level top-down processes were targeted by investigating if priming was location-invariant and cue-invariant with respect to our three prime conditions: a disambiguated SFM prime, a pictorial prime, and an imagery prime (which is entirely endogenously generated). Additionally, we examined whether individual differences in priming, reversals and mixed percepts of our ambiguous SFM-stimulus were related, as suspected if a common disposition to change perceptual interpretation is involved (e.g. the activity of a hypothesis generator attempting to explain sensory signals). In addition, inspired from previous reports of correlations between personality traits and occurrences of reversals from other ambiguous stimuli, we sought to find such relations with our ambiguous SFM stimulus using our results from priming, counts of reversals and mixed percepts.

There are many types of ambiguous stimuli: The Necker cube, a static parallel projected wire frame cube with two possible depth interpretations was first presented in 1832 [1], and in 1838 Wheatstone described binocular rivalry where dissimilar images presented to the left and right eye results in perception of spontaneous switches between these images [2,3]. Rubin’s face-vase, presented in 1942 [4], is another classic example of ambiguous perception involving figure-ground relations. Structure-from-motion (SFM), first demonstrated in 1953 [5] is typically displayed as parallel projected revolving spheres or cylinders, where only dots on the surface of the object are visible and the perceived rotation direction is ambiguous. The perception of such ambiguous figures can be influenced in various ways, for example, the fixated part of the Necker cube tend to appear as the front side [1,6–8], and since fixation determine the input to the visual system it can be regarded as an instance of bottom-up driven influence on perception [9]. Also prior experience and even volition can influence the relative dominance of ambiguous perceptions, although perceived spontaneous reversals during prolonged viewing are hard to prevent [10,11]. By relating perception of ambiguous figures and neural activity it is also possible to probe neural correlates of consciousness [12,13]. For example, in the case of the face-vase figure, activity in face-area increases when the face percept emerges [14]. Also, the visual processing underlying spontaneous perceptual reversals of rotation direction while observing an ambiguous SFM stimulus is accompanied by changes in connectivity strength between parietal and visual cortical regions [15]. A number of theoretical proposals have been made as to the nature of perceived reversals while viewing ambiguous stimuli, such as the activity of a top-down driven generation of hypotheses to explain sensory data [16,17] or low-level turn taking due to adaptation and inhibitory connections between competing neural circuits [18,19]. There is, however, no ultimate consensus on how prior experiences influence the perception of ambiguous stimuli, what drives the switches between percepts, or why people experience widely different reversal rates.

Interestingly, the switching rates of ambiguous rotation direction of a SFM stimulus are not related to switching rates of the Necker cube, the face vase figure-ground stimulus, or binocular rivalry, suggesting that different or independent processes mediate switching rates [20]. In addition, there is a qualitative difference between figural ambiguities, such as the Necker cube or SFM, and binocular rivalry. In figural ambiguities, all sensory input can be explained by the current, dominant percept. In binocular rivalry, on the other hand, the visual inputs from one eye must be entirely suppressed. Thus, the lack of correlation between switching rates from various ambiguous stimuli and the qualitative difference between binocular rivalry and figural ambiguities may indicate that research on binocular rivalry and Necker-like stimuli may not be generalizable to ambiguous SFM stimuli.

### Perceptual ambiguities and low-level processes in SFM

Ambiguous SFM is typically studied by simulating revolving spheres or cylinders. The few studies that have investigated priming of such ambiguous SFM stimuli suggest that it involves low-level bottom-up driven processes. For example, a sequential presentation of a disambiguated rotating SFM-prime followed by an ambiguously rotating SFM-test stimulus, with a delay in the order of seconds, results in priming of the perceived direction of rotation of the test stimulus: it tend to be perceived as rotating in the same direction as the prime. Such priming involves short-term motion-memory traces and is independent of attention [21,22]. Efficient priming of rotation direction occurs between identical SFM objects but not dissimilar objects [22] (but see [23]). Priming of object detection from a noisy SFM stimulus occurs when the prime consists of an SFM specifying the same object without noise, but not when the prime is an identical static object, or when a semantic word describes the same object [24]. In addition, there is evidence for strong fusion between SFM and stereopsis at early stages of processing: First, presenting dots in motion in opposite directions at stereoscopically separated depth planes behind and in front of an ambiguously revolving SFM globe results in successful disambiguation of the globe, being aligned with the stereoscopic motion [25]. Second, adaptation to an SFM globe disambiguated by stereoscopic cues cause a subsequently presented ambiguous globe to rotate in opposite direction to the adaptor, but no adaptation effects occur when other cues are used to disambiguate the direction of rotation [26,27].

Perceived reversal rates of a revolving SFM stimulus drop remarkably by frequent alterations of the simulated rotation direction, probably due to interrupted low-level adaptation to direction [28]. The sign of slant signalled from velocity gradients in parallel projections of SFM is also ambiguous with respect to the sign of slant and the percept may appear to switch spontaneously back and forth between forward and backward slant [29]. When the slant oscillates little back and forth during observation, the frequency of spontaneous switches between the perceived sign of slant is also drastically reduced [28]. These results suggests that during oscillations, adaptation of low-level motion direction and orientation selective units is hampered, slowing down perceived rates of reversals caused by adaptation and recovery between competing turn taking neural circuits [28]. Taken together, the findings exemplified above are hallmarks of low-level bottom-up driven processes involved in the perception of ambiguous SFM stimuli.

### Perceptual ambiguities and high-level processes

Helmholtz [30] wrote in 1866 that he could exert full control over the perceived interpretation of ambiguous stimuli by paying attention to either interpretation of it. This is a top-down driven process influencing perception (for an opposing view arguing that perception is immune to top-down processes, see [31]). Visual mental imagery, perceiving things before the mind’s eye, plays an important role in in generative models such as the “Helmholtz machine” [32]. In generative models, such as the recently formulated predictive processing, sensory information is analysed by comparing it to endogenously generated similar patterns [33,34]. Influences of such endogenously generated imagery on perception have been reported using ambiguous figure-ground stimuli [35] and binocular rivalry [36]. Other evidence for the involvement of top-down processes in perceptual switching during binocular rivalry is the accompanied responses in ventral regions that precede those in early visual regions, which is reversed compared to processing of bottom-up driven stimulus changes [37]. In a cross modal associative learning task the ambiguous rotation direction of an SFM stimulus can be selectively manipulated using sounds that observers learned to associate with either direction of rotation [38]. The Necker cube ambiguity can be influenced by flanking the Necker cube with fields of unambiguous cubes that are oriented to coincide with one of the Necker cube percepts [39]. For both the ambiguous Necker cube and the face-vase figure, both top-down and bottom-up influences are elevated in observers during viewing as compared to viewing of stable versions of these figures; top-down influences do however outweigh bottom-up influences during perceptual switching [40].

Knowledge of reversibility and instructions to switch between alternative perspectives as quickly as possible are both known to increase reversal rates [41–43], and tasks which divert attention away from the ambiguous stimulus decrease reversal rates [41], even if the task at hand is non-visual [44]. Voluntary control of the perception of reversible figures has been demonstrated, and control over one type of ambiguous figure is highly correlated with the control over another type, suggesting the presence of stable individual differences in ability to control perception voluntarily [11]. Interestingly, this is contrary to the lack of such relations for switching rates between different ambiguous stimuli [20]. Voluntary control is, however, less efficient during binocular rivalry, which is believed to involve a more automatic, stimulus-driven form of visual competition than Necker cube reversal [45]. Conversely, when Horlitz and O’Leary [35] trained their subjects to imagine one interpretation of figure-ground reversible figures, the same interpretation was favoured upon exposure to the reversible figure suggesting that priming occurred from imagery alone.

Cross modal influences, influences of context, and brain imaging studies demonstrates that a local process based solely on bottom-up driven turn taking due to habituation and inhibition is insufficient to account for all phenomena involved in the perception of ambiguous stimuli.

### Perceptual ambiguities, personality, and cognitive modes

A few studies have investigated possible relations between personality traits/cognitive abilities and perception of ambiguous figures. Such relations could be a result of general top-down driven processes or result from local variations in neurotransmitter activity influencing both perception and personality traits. For example, anxiousness, a marker for the Big-5 trait neuroticism has been linked to higher switching rates when viewing binocular rivalry stimuli [46], and Nagamine et al. [47] found that a highly selective agonist to a specific serotonin receptor commonly prescribed as an anti-anxiety drug, slowed down switching rates. Also, a positive correlation has been reported between intelligence and switching rate from binocular rivalry [48]. Antorini et al. [49] hypothesised that traits such as flexible and inclusive cognition, characteristic for people scoring high in the openness to experience trait in the Big-5 questionnaire may extend to basic visual perception, such that open people may combine sensory information more flexibly. Interestingly, their hypothesis was confirmed as they found a positive correlation between the Big-5 trait openness and the occurrences of mixed percepts during binocular rivalry, meaning that both versions of the rivalry stimulus are seen at once [49]. No correlation, however, was found between openness and reversal rates during binocular rivalry [50]. Mixed percepts and divergent thinking, found in persons high in openness [51], such as coming up with multiple uses for an object may be driven by a common process: an individual variability in the details (or granularity) of the hypotheses formulated in an attempt to find multiple uses for an object, or to explain incoming sensory data [52]. If so, the correlation between openness and the frequency of mixed percepts should generalize to other ambiguous stimuli than binocular rivalry.

Other trait dimensions have also been linked to reversal rates during observation of ambiguous stimuli. Lower reversal rates in binocular rivalry seem to be associated with higher levels of self-discipline (one of the six facets of conscientiousness in Big-5) [53]. Lower reversal rates for introverts than extroverts are reported in studies using figure-ground ambiguity [54], binocular rivalry stimuli, and the “wind-mill illusion” [55] (consisting of a shadow of a rotating wheel obliquely projected on a screen that can be perceived as rotating clockwise or anti-clockwise). Contrary to these studies, higher reversal rates for introverts have been obtained with a three-dimensional reversible figure [56]. Some studies have reported that clinically diagnosed autistic people experience lower switching rates during rivalry [57–59] (but see [60]) and lower reversal rates when observing Necker cubes [61] and ambiguously revolving SFM-stimuli [62].

Although previously not investigated, the influence from priming of ambiguous figures might be related to people’s cognitive styles. Pacini and Epstein’s [63] cognitive-experiential self-theory (CEST) propose that people make use of two different cognitive styles; rational and experiential. Rauss and Pourtois [64] describe the use of fast heuristics as a top-down process in an experiential style, that bypass applying rules to bottom-up data in a slow rational style, thus providing a possible link between cognition and perception in terms of bottom-up and top-down processes.

### Aims

Our primary aim was to target top-down influences in priming of a SFM-test cylinder whose orientation in depth is ambiguous while its orientation and rotation oscillates back and forth with small amplitudes to result in wiggling motion. By using this SFM stimulus influences of low-level processes believed to be involved in ambiguous SFM perception [21,22,24–28] are minimised and we can address questions focusing on aspects of high-level processes. In particular, is priming location-invariant and cue-invariant? Such invariances are characteristics of higher-level processes. In addition, if the underlying process is identical across cues used to specify the prime then influences from the different primes should be equal and equally correlated (Experiments 1 and 2). Our secondary aims were to investigate possible associations between perception of the ambiguous SFM stimulus as measured with priming, reversal rates, and mixed percepts, and these perceptual measures and personality trait dimensions. Watanabe et al. [62] included a response option labelled mixed percepts to use by the participants when they could not distinguish a single stable rotation direction, while viewing an ambiguously revolving SFM sphere (in investigating the relation between autism and duration of stable percepts). We hypothesise that if there is a common factor involved, such as proneness to change interpretation of incoming stimuli (i.e. due to the activity of a common hypothesis generator) correlations will be found between priming, number of reversals, and mixed percepts. Although previous research has demonstrated specific relations between switching rates for some ambiguous stimuli and personality, relations between priming and personality dimensions has not yet been investigated.

Specific predictions follow from previous research examining relations between personality traits and ambiguous perception, mainly from using binocular rivalry stimuli and the Necker cube. Specifically, can the previously found positive correlation between reported mixed percepts during binocular rivalry and openness found by Antinori, Carter, et al. [49] be reproduced using our ambiguous SFM stimuli when mixed percepts is included as a response option? Initially we hypothesised that among all Big-5 dimensions, the openness dimension to be most likely associated to individual variability in influences of top-down processes, and to be correlated with top-down mediated priming. Therefore, in Experiment 1, only the questions for the openness dimension in the Big-5 inventory [65] was included together with the vividness of visual imagery questionnaire (VVIQ) [66] scoring the vividness of endogenously generated images, targeting specific high level process that could potentially influence perception. Based on the results from Experiment 1, in Experiment 2 we included all five traits in the Big-5 personality questionnaire. In addition we included vividness of imagery as scored by the Vividness of Object and Spatial Imagery questionnaire (VOSI) [67] that provide separate scores for endogenously generated object and spatial imagery, autism spectrum scores from the Autism spectrum Quotient questionnaire (AQ) [68], and cognitive styles as scored by the Rational Experiential Inventory questionnaire (REI) [63].

Based on Antinori, Carter, et al. findings we hypothesise that the number of reported mixed percepts while observing our ambiguous SFM stimulus correlates positively with scores on the Big-5 trait openness (Experiment 2). We also explored if we could find a correlation between openness and the influence from top-down mediated SFM-priming (Experiment 1 and 2). Anxiousness, which is linked to the trait neuroticism, has been shown to correlate with switching rates during binocular rivalry [46]. Hence, we predict that the Big-5 trait neuroticism is positively correlated also with reversal rates while viewing our ambiguous SFM stimulus (Experiment 2). Both reduced reversals [61] during observation of Necker cubes and switching during binocular rivalry [58], and no influence in switching rates during binocular rivalry [60] have been reported for those clinically diagnosed with autism. We use our ambiguous SFM stimulus to explore the relation between reversals and scores from the AQ inquiry on our non-clinical sample (Experiment 2). Also of interest is the question of whether susceptibility to priming, reversals, and mixed percepts are correlated, which is likely if they have a common origin, for example a general flexibility in interpretation of sensory input (Experiment 2).

Novel to our knowledge and not based on previous results are specific predictions involving relations between priming and visual imagery as scored with VVIQ and VOSI. We expect that high level priming (particularly the influence of imagery priming) correlate with imagery as scored by the self-rating questionnaires (VVIQ in Experiment 1 and VOSI in Experiment 2). Also novel are predictions involving relations between priming and cognitive styles as scored by REI. The experiential cognitive mode can be characterised as a top-down process providing fast experience based decisions, whereas the rational mode requires application of rules and logic to operate the bottom-up flow of input to arrive at a decision. Is it possible that the perceptual top-down process involved in priming and cognitive top-down flow of information as scored by REI tap the same underlying processes (Experiment 2)? If so, we expect that scores on the experiential top-down driven mode are positively correlated with the influence of top-down driven priming, whereas scores on the rational bottom-up driven mode are negatively correlated.

## General methods

**Fig 1:**
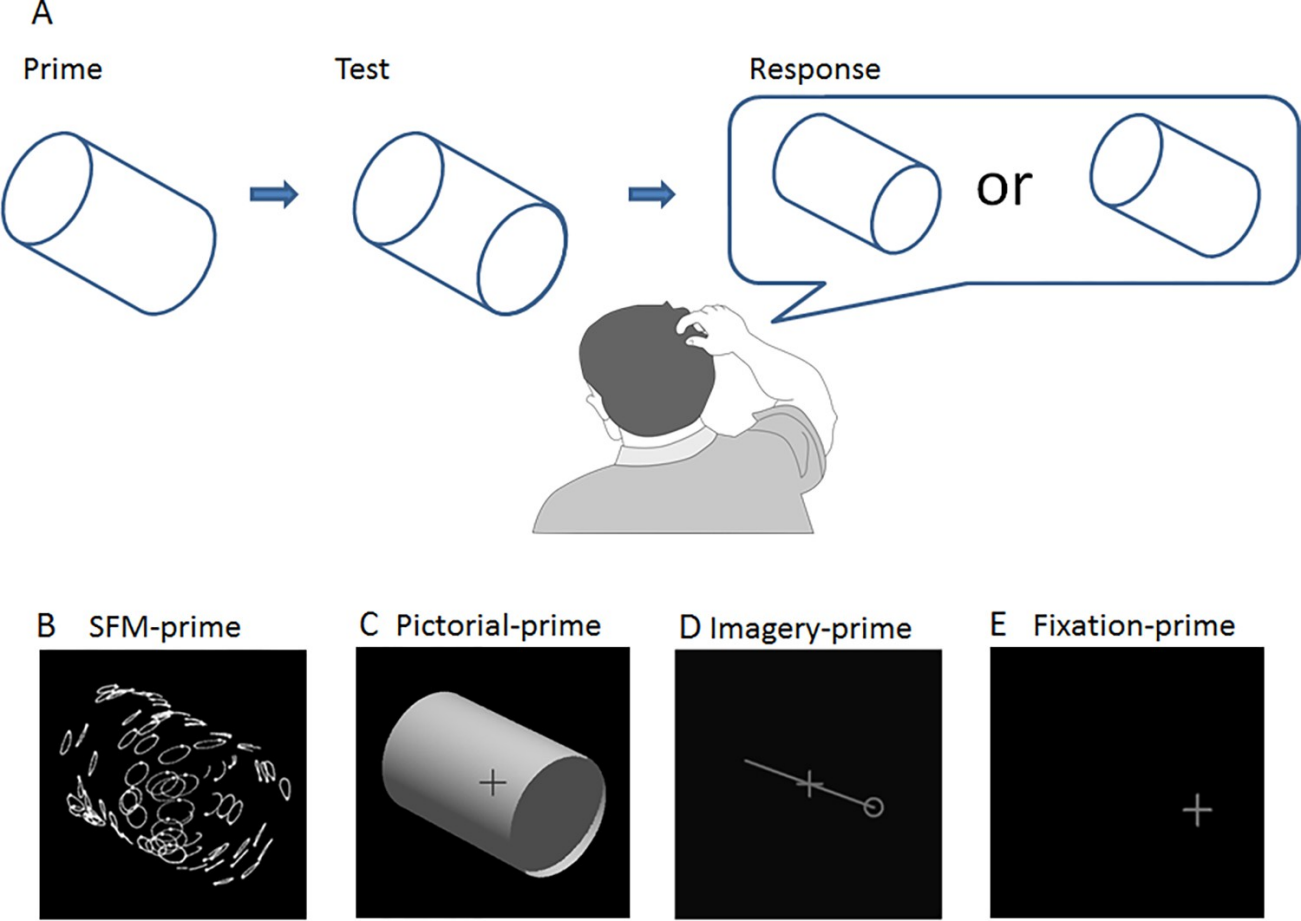
The priming sequence and the primes. A. Priming results in reporting the ambiguous test cylinder as having the same orientation as the stable prime cylinder. In Experiment 1 primes B to E were used and in Experiment 2 primes B to D were used. B. Structure-from-motion prime-cylinder, a non-transparent cylinder where dots are occluded by the near side of the cylinder, displayed with a long exposure showing a complete period of the motion strikes of individual dots. C. The pictorial prime-cylinder, revealed by shading due to illumination from above. D. An example of the imagery prime, with the orientation and opening of the cylinder to be imagined hinted at by the line and the circle. E. Fixation condition where only the fixation cross appeared at one of the openings of the upcoming test cylinder.

The general idea behind priming as applied here is to first present a stable version of the cylinder, the prime, and then after a brief blank period to present the ambiguous SFM cylinder, the test stimulus. The observers’ task is to report in each trial which interpretation of the test cylinder is perceived (Fig 1A, see demo SFM-test, S1 Movie). Priming then results in observers reporting the orientation of the test cylinder to be the same as that of the prime cylinder. To investigate cue-invariance and to pin down higher-level processes involved in priming we used two visually displayed prime-cylinders. In one condition, the prime was a stable SFM cylinder where occlusion through accretion and deletion of dots during the motion sequence disambiguated its orientation. Fig 1B is a long exposure image of a motion sequence covering a whole motion period of a SFM-prime with its right side opening pointing toward the observer, and tilting downward (see demo, SFM-prime, S2 Movie). Another prime consisted of a pictorial still image of a cylinder specified by luminance cues such as shading (Fig 1C). In the endogenous prime condition, observers were instructed to imagine a prime cylinder with a specific orientation. This was cued by a line with a circle at one of its ends to indicate the orientation of the cylinder to be imagined (Fig 1D). In Experiment 1 a fourth condition was included where the influence of fixation was investigated (Fig 1E). Initially, the purpose of the fixation condition was to make sure that if priming was found it would not primarily be a result of observers having directed their gaze or covertly directed their attention to the closest part of the prime and then held their gaze or covert attention on the same spot when the test cylinder showed up. From introspection, we suspected that the primes would be far more efficient than gaze direction in influencing the perceived orientation of the ambiguous test cylinder. The results did not support this suspicion. Therefore, in Experiment 2 we presented the ambiguous test cylinder at a randomly chosen location along a circle at a fixed distance from the prime while observers were instructed to fixate a fixation cross at the middle of the display during the whole trial. This prevented the influence of fixation or covert attention. By comparing priming between Experiment 1 and 2, possible position invariance could also be investigated. Additionally, in Experiment 2, each participant counted perceived reversals, and mixed percepts (when it was not possible to distinguish any stable percept of the test stimulus, as defined in a previous study [62]) during five minutes of observation of the ambiguous test cylinder.

Responses in the priming experiments were made by pressing the F and K-keys to indicate whether the left side or right side of the test was perceived closest. Responses to indicate perceived reversals were also made by pressing the F and K-keys to indicate left side closest or right side closest, and the space bar to indicate mixed percepts during the 5 minutes observation.

### The structure-from-motion prime and test cylinder

**Fig 2:**
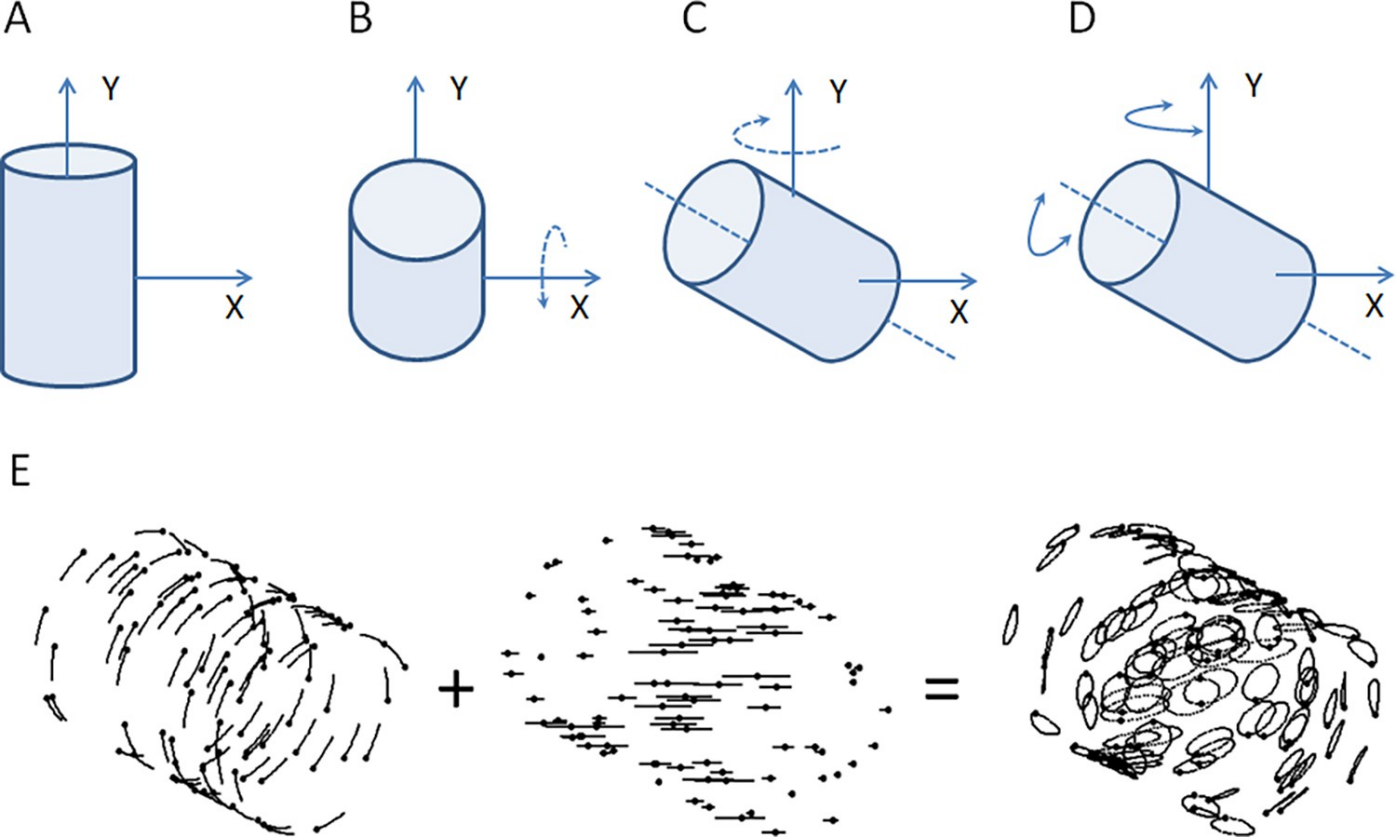
Construction of the SFM cylinder. Dots were randomly spread on the surface of an invisible cylinder. (A) Initially, dots were spread on the cylinder with its main axis oriented along the vertical y-axis. (B) To obtain the initial orientation of the cylinder it was slanting around the x-axis (C) and then tilted around the y-axis. (D) From the initial orientation, small steps of rotation around the cylinder main axis and around the vertical y-axis between each frame created the wiggling motion. (E) Adding these two oscillatory rotation components created elliptic motion paths of the individual dots, here visualised as motion strikes obtained from a long exposure.

**Fig 3:**
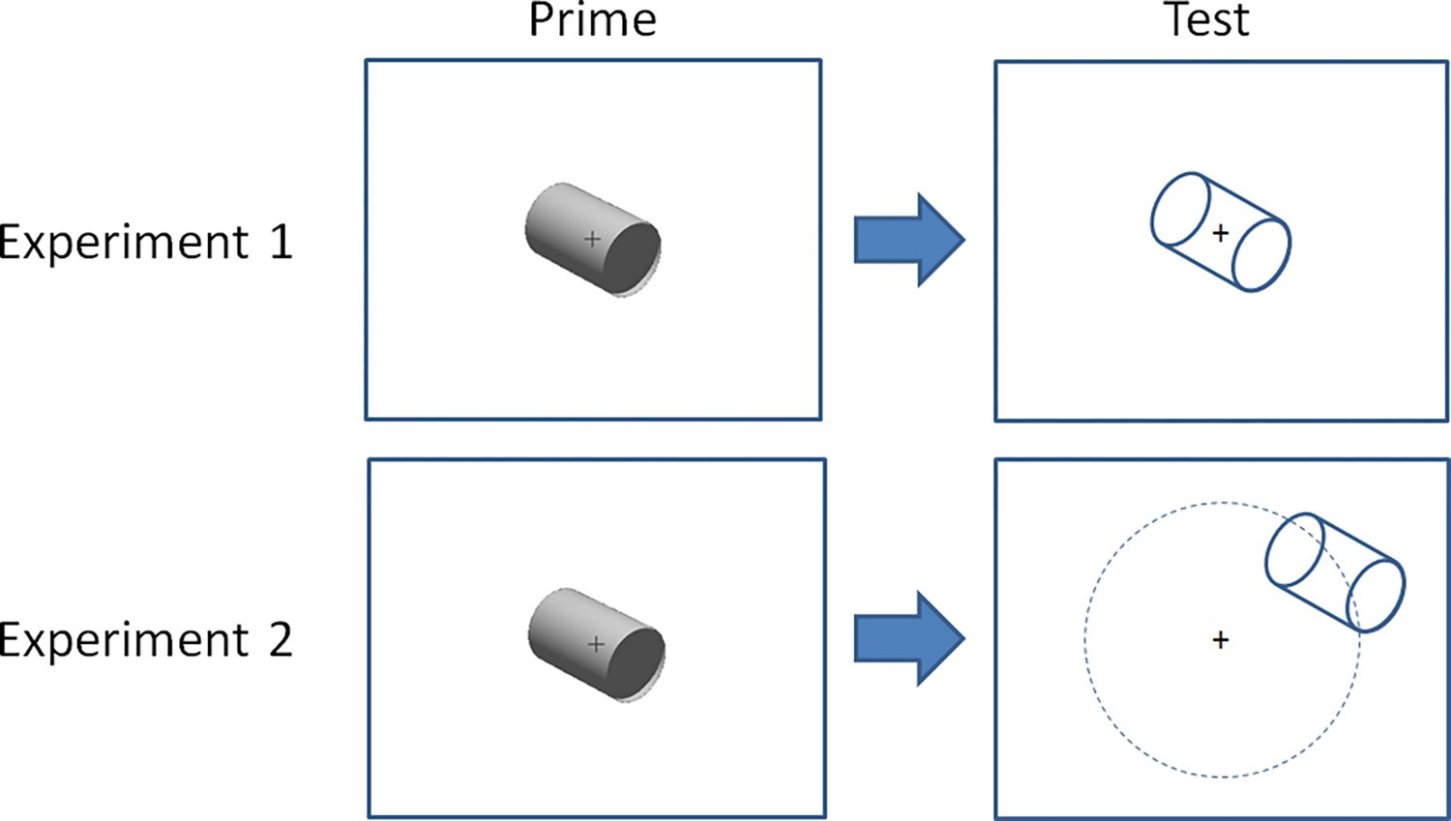
Priming in Experiment 1 and 2. In Experiment 1, the test stimulus was located at the same position as the previously presented prime. In Experiment 2, the test stimulus was randomly located along a circle with a fixed radius around the prime.

The SFM-prime was created by randomly spreading 500 dots across the surface of a simulated cylindrical tube (radius 2.5 cm and length 7.5 cm on the screen) aligned with the vertical y-axis (Fig 2A). Each dot location in a cylindrical coordinate system was randomly chosen (an angle between 0–360 deg and a y-coordinate between the endpoints of the cylinder). To be able to apply the formula for rigid rotations the cylindrical coordinates were transformed to Cartesian coordinates. The x-axis was directed radially to the right from the midpoint of the cylinder and the z-axis directed toward the observer. Rigid rotations were performed sequentially as shown in Fig 3A to 3D. The cylinder was first slanted + 70° or—70° around the radial x-axis resulting in a forward or backward slant (Fig 2B). Next, the cylinder was tilted + 45° or—45° around the vertical y-axis (Fig 2C) resulting in a sideway tilt as seen from the observers perspective. From this +/- 70° slant and +/- 45° tilt position the cylinder was set into its oscillatory rotation composed of the sum of two components: an oscillatory rotation around the cylinder main axis synchronised with an oscillation around the vertical y-axis (Fig 2D). The angle between frames of both rotations were modulated sinusoidally to create the wiggling motion of the cylinder to reveal its shape and minimise adaptation to direction and slant that could occur if direction and slant were not oscillated [28]. Both oscillation components had identical periods of two seconds, and equal amplitudes +/-10° (Fig 2D). A complete motion period consisted of 50 sequentially presented movie frames created by parallel projecting all dots onto the image plane (the computer screen) parallel to the x-y plane. The motions of individual dots, resulting from combining these two rotations, appeared as elongated closed paths on the projection plane (Fig 2E). This oscillatory motion minimise priming and adaptation of low-level motion-direction selective units [28].

The ambiguous version of the cylinder (the test cylinder) was transparent so all dots on both the front and back were visible throughout the motion sequence without being occluded. The cylinder was slanted in depth and perceptually ambiguous with respect to the sign of slant. Either it could be perceived with its left or right opening directed toward the observer (demo SFM-test, S1 Movie). The perceptually stable disambiguated SFM cylinder used as one of the prime stimuli consisted of an opaque cylinder involved in wiggling motion identical to the motion of the test-cylinder, but with its front surface occluding its back surface (demo SFM-prime, S2 Movie). During the motion sequence, as dots on the back surface of the cylinder were covered and uncovered by the occluding front surface they were deleted and accreted, which is a strong local cue to the depth relations. In each trial, the prime-cylinders were presented with combinations of clockwise and anticlockwise tilts (+/- 40°) and forward and backward slant (+/- 70°) in random order. The test-cylinder was always presented with the same tilt as the prime-cylinder whereas its slant was ambiguous.

### The pictorial prime cylinder

Realistic still images of the pictorial-prime (Fig 1C) were created by a simple raytracing algorithm, where virtual light-rays are followed back from the observers’ vantage point to each spot on the surface of the cylinder. On each spot where the line of sight crossed the cylinder, the angle between the traced ray and the cylinder surface normal, as well as the direction from the spot on the cylinder to the source of illumination, was calculated. Where the line of sight crossed the cylinder twice, only the front surface was visible due to occlusion. If a straight line from the source of illumination to a visible spot on the cylinder hit a part of the cylinder that shaded that visible spot, then that spot was coloured with dark grey to indicate that it was shaded from direct illumination. The reflectance and light spreading properties of the cylinder were defined by fixed parameters and the direction vector pointing to the light source above the cylinder was fixed. The luminance at directly illuminated spots of the cylinder was calculated from these parameters together with the direction vector pointing to the observer and the cylinder surface normal vector at each spot on the cylinder surface.

### Imagery prime and fixation prime

In the imagery-priming condition, observers were instructed to imagine a cylinder with a specific orientation in 3D-space from a cue consisting of a line with a circle on one of its ends. Observers were to imagine the cylinder as having its opening at the location of the circle and to be oriented along the presented line (Fig 1D). Influence of gaze direction in Experiment 1 was investigated by instructing observers to fixate a cross at either end of the subsequently presented test cylinder before it appeared. The instruction was to keep fixating that spot when the test cylinder appeared (Fig 1E).

### General procedures

The priming sessions were performed in a lab with weak dimmed light. Stimuli were presented on a 24-inch screen with 1920 x 1200 resolution, 60 Hz refresh rate, and 32-bit colour. Observers eye positions were about 60 cm distance from the screen so 1 cm on the screen corresponded to about 1 degree of visual angle. Examples of prime and test stimuli were shown prior to the priming. The prime and test was presented for 2000 msec (allowing for a complete motion period for the SFM cylinder) while a green fixation cross was presented in the middle of the screen. The delay between prime and test was also 2000 msec. In the priming experiments participants responded by pressing the F or K keys (colour coded to avoid confusion) on the keyboard to indicate whether the left or right part of the test cylinder was perceived as closest. To initiate the next trial they pressed the spacebar. Participants were allowed small breaks between trials and between the different parts of the study, as well as between different priming conditions. In Experiments 1 and 2, the prime conditions were blocked and balanced between participants. Each priming block contained 52 trials presented in randomised order. The priming in Experiment 1 took about 60 minutes and in Experiment 2 about 45 minutes.

In Experiment 1, the prime and test cylinders were presented at the same location on the centre of the computer screen (Fig 3). After the priming sessions, paper copies of questionnaires with 26 items were administered to the participants. These included the vividness of visual imagery questionnaire (VVIQ) [66], rating the clarity and liveliness of visual imagery. It contain 16 items responded to by using a five point rating scale (ranging from “No image at all, you only “know” that you are thinking of an object” to “Perfectly clear and as vivid as normal vision”, in Swedish). Also included in the questionnaire was questions related to the personality trait openness (10 items), which can be described as a general receptivity to new ideas, appreciation for imagination, curiosity, and variety of experience, were taken from the Swedish Big-5 [65] personality test. The questions were propositions of personality characteristics and responses were made using a five point rating scale (ranging from “absolutely not” to “absolutely true”).

In Experiment 2, the test cylinder appeared at a random location along a circle with radii 5.4 cm around the prime (Fig 3). After the priming session in Experiment 2, we measured the perceptual switching rate between the two rigid interpretations of the ambiguous cylinder and the occurrences of mixed percepts. Key presses on the F and K-keys indicated left closest or right closest respectively, and pressing the space bar indicated mixed perception. Finally, in Experiment 2, paper copies of questionnaires were administered including the complete Big-5 inventory with all five personality dimensions (Swedish version [65], with 44 items scoring for openness, conscientiousness, extraversion, agreeableness, and neuroticism), and autism quotient (AQ, [68], with 50 items) measuring the degree to which an adult with normal intelligence has the traits associated with the autistic spectrum. Also included was the vividness of object and spatial imagery questionnaire (VOSI, [67], with 28 items) providing separate scores for object and spatial imagery, and the rational experiential inventory (REI, [63], with 40 items) which assesses tendencies to engage in rational and experiential information processing, which are conceptualized as distinct thinking styles. Rating scales were used for each of the 162 items in Experiment 2, and scored as described in the test instructions for each test.

### Statistical analyses

Statistical tests were performed by the freely available statistical software JASP [69] to calculate frequentist p-values and Bayes factors. The Bayes factor BF_10_ is the ratio between the probabilities of the results given H_1_ and H_0_ and is thus a measure of the relative evidence in favour of H_1_. Bayes factors are considered more intuitive than p-values and have several other advantages [70]. For example, unlike the p-value, the Bayes factor can be interpreted as evidence in favour, or against, the null hypothesis (H_0_). All reported Bayes factors were computed using the default settings in JASP for the likely prior distribution of effect sizes under H_1_ (for correlations this is the uniform stretched beta prior corresponding to a uniform prior distribution of correlations). Varying the width of the prior distribution within reasonable limits has small influences on the BF [71]. As a guideline it has been suggested that BF_10_ (BF_01_) between 1 and 3 (1-1/3) should be considered as anecdotal evidence, from 3 to 10 (1/3–1/10) as moderate evidence, from 10 to 30 (1/10–1/30) as strong evidence, between 30 to 100 (1/30–1/100) as very strong, and beyond that extremely strong evidence [72]. Since Kendall’s rank correlation coefficient tau-b is recommended when samples are small or when many values with the same score (ties) are found, and Bayes factors for Tau-b could be calculated in JASP, this option was chosen in the analyses of reversals and mixed percepts in Experiment 2. Parametric tests were otherwise used (no difference in conclusions were reached by switching from parametric to non-parametric tests, or the other way around). Testing for differences between two correlation coefficients were performed using a frequentist two tailed test [73] available on-line at http://quantpsy.org/corrtest/corrtest2.htm (no Bayes factor estimate available).

### Ethical considerations

Consent to participate in this study was obtained in written form from each participant. They were informed that their participation will be completely anonymous so their identity would not be revealed, and that they could cancel the experiment at any time with no consequences. The studies were approved by the Regional Ethical Review Board in Uppsala and were conducted in accordance with the Declaration of Helsinki.

## Experiment 1

The prime and test cylinders were presented sequentially at the same location on the computer screen. Four prime conditions were used: a disambiguated SFM-prime cylinder (Fig 1B), a still image of a cylinder (Fig 1C), an imagined cylinder with a specific orientation (Fig 1D), and to examine influences of fixation a fixation-cross presented at one of the ends of the ambiguous test cylinder shortly before it appeared (Fig 1E). Questionnaires with items from the VVIQ and the openness dimension of the Big-5 personality test were administrated after the priming experiment.

### Participants

The average age of the 82 participants included in the analyses (46 female and 36 male) was 27 years (Std = 11). Four other participants were excluded from further analyses due to interrupted tasks. They were mainly recruited from the campus and Facebook and were given a cinema ticket for compensation.

### Results

**Fig 4:**
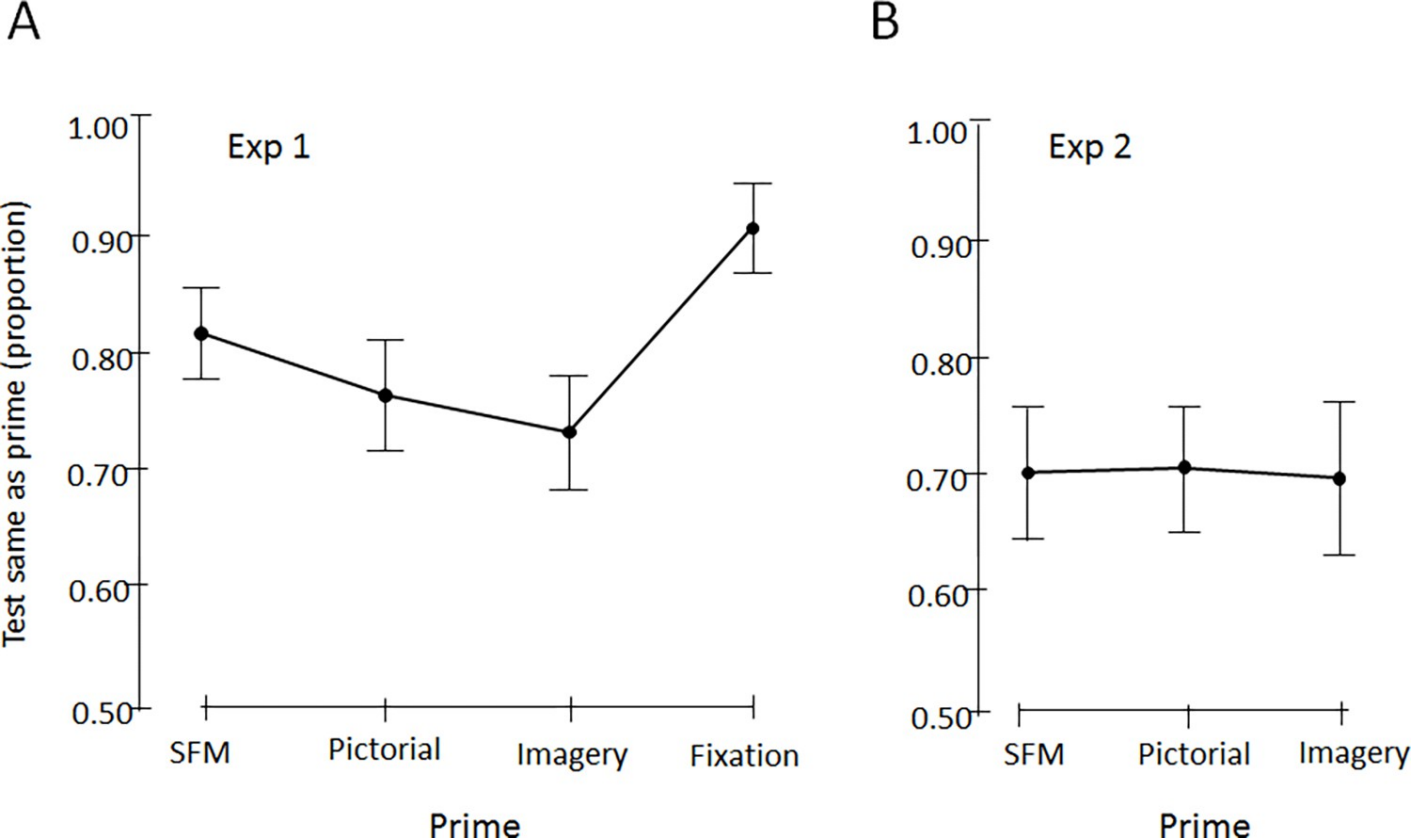
Mean priming. The graphs show the proportion of responses where the orientation of the ambiguous test cylinder was perceived as the same as the prime cylinder. The .5 level indicates no relation between the orientation of the prime and perceived orientation of the test stimulus. (A) The average influence of the primes from Experiment 1 where the prime and test were located at the same place, and a fixation condition where no prime was presented. In the fixation condition, before the cylinder appeared participants were instructed to direct their gaze to a location where one of the ends of the ambiguous cylinder would appear. The proportion indicates occurrences where the gaze direction coincides with the perceived near part of the ambiguous cylinder. (B) In Experiment 2 the test cylinder appeared at a random location around the previously presented prime to prevent influences of cueing to the nearest perceived part of the prime. The 95% CI’s are displayed.

Fig 4A shows the mean influence of the prime across all observers quantified as the proportion of test cylinders reported as having the same orientation as the prime. Proportions over .5 indicates priming and proportions below .5 mean that the test cylinder in most trials was perceived as having the opposite orientation compared to the prime.

Priming was obtained in between 73 to 91% of trials, where the strongest influence was obtained from the fixation condition. Table 1 shows the results from one-sample t-tests used to infer if sample average differ from a population value of 0.5, and paired samples t-tests to infer if pairwise differences are different from zero. The only non-significant difference was found between the pictorial and imagery priming, although the support for a true absence of a difference, with a BF_10_ = .53 is considered anecdotal.

**Table 1:** Influence of primes and differences between primes in Experiment 1.

*Prime conditions*	t(83)	p	BF_10_*	Cohens’ d
SFM	16	< .001	3.1·10^24^	1.80
Pictorial	11	< .001	4.8·10^14^	1.19
Imagery	9.4	< .001	5.7·10^11^	1.02
Fixation	21	< .001	1.4 · 10^32^	2.33
*Differences*	t(82)			
SFM-pictorial	2.6	.01	3.2	.29
SFM-imagery	4.2	< .001	333	.47
Pictorial-imagery	1.8	.081(ns**)	.53	.19
Fixation-SFM	3.9	< .001	113	.43
Fixation-pictorial	5.8	< .001	1.5·10^5^	.64
Fixation-imagery	6.8	< .001	9.0·10^6^	.75

One-sample t-tests applied to the results from Experiment 1 investigating influence of the different primes. Also shown are analyses of the differences between conditions from paired sample t-tests. All tests are two-tailed.

* The default Cauchy prior, with width parameter .707, was used.

** Non-significant difference.

The very strong influence from the fixation condition where the perceived closest side of the test cylinder coincide with the direction of gaze was contrary to expectations. This may indicate that priming was also affected by fixation where observers could not resist directing their attention, overtly or covertly, toward the closest side of the prime cylinder and kept it at the same spot when the test-cylinder appeared.

If gaze direction, or covert attention, was a dominant cause to the influence from the primes acting as a fixation cue (similar to the fixation cross in the fixation condition), and this effect varied between individuals, then a strong correlation is expected between the influence from gaze in the fixation condition and influence from gaze in the prime conditions. Table 2 shows the correlations between the influences of the primes. We present Pearson´s r but the same results and conclusion was reached with non-parametric correlations. Stronger correlations were found for the pairwise prime conditions not involving fixation (.59 < r < .73, between 35–53% shared variance) than the correlations involving fixation (.16 < r < .32, between 2.6–10% shared variance). This provides evidence that fixation is a minor cause to the influence from the primes.

**Table 2:** The Pearson correlations between priming conditions in Experiment 1 (two tailed tests, N = 82).

Pairs of Prime conditions	r	p	BF_10_
SFM -Pictorial	0.59	< .001	2.1·10^6^
SFM- Imagery	0.61	< .001	1.1·10^7^
Pictorial - Imagery	0.73	< .001	1.6·10^12^
SFM- Fixation	0.16	.148	.385
Pictorial- Fixation	0.32	.003	10
Imagery- Fixation	0.31	.005	7.2

The correlation between the SFM and pictorial priming, and the correlation between the SFM and imagery priming are weaker than the correlation between the pictorial and imagery priming (p < .05, testing for difference between correlations [73]). Also, the BF supporting a correlation between the pictorial and imagery priming (BF_10_ ≈ 10^12^) is more than 100 000 times greater than the BF supporting correlations between SFM and pictorial priming (BF_10_ ≈ 10^6^), and between SFM and imagery priming (BF_10_ ≈ 10^7^). This indicates that some other process is involved in the priming when both the prime and test are defined by SFM (SFM prime-SFM test) as compared to cross-cue priming (pictorial prime-SFM test, and imagery prime-SFM test). This SFM specific process disrupts the correlation between the SFM prime and the other prime conditions. One such possible process is a cue specific low-level priming activated by the SFM-prime as opposed to the cross-cue priming. Another possibility is the higher visual similarity with the SFM test stimulus, whereby prime and test are perceived as the same object, which in principle could be attributable to a higher-level process.

We also let the participants fill in the vividness of visual imagery questionnaire (VVIQ) and selected questions of openness in the Big-5 personality questionnaire. No correlation between priming and openness were obtained (-.14 < r < -.056, all p’s >.05; .15 < BF_10_ < .28 meaning that the results given the absence of correlation are between 3.6 and 6.7 times more likely than obtaining the results given that there was a correlation). Interestingly, no correlations between VVIQ-scores and influence of priming were found, not even the imagery priming condition (.034 < r < .11, p’s > .05; .14 < BF_10_ < .22 meaning that the results given the absence of correlation are between 4.5 and 7.1 times more likely than obtaining the results given that there was a correlation). Although not part of our research questions we found that openness and VVIQ scores were correlated (r = .42, p < .001, BF_10_ = 280).

The data set from Experiment 1 is provided as supporting information (S1 Data, in sheet 1).

## Experiment 2

Some residual influences of gaze, or covert attention, may underlie the 10% shared variance between influences of the test-stimulus from the fixation condition and prime conditions in Experiment 1. There is also a possibility that the influence of the prime may have been specific to the retinotopic location where the prime was presented. Such retinotopic specificity is a sign of low-level processes. In Experiment 2, we wanted to minimise these possibilities: The prime still showed up in the centre of the screen, but the test cylinder appeared at a random location at a fixed radius 5.4 cm from the centre of the prime where the fixation cross was located. This considerably reduces the risk of influences of gaze direction, or of covertly directing attention to specific locations on the screen, and minimised retinotopic specificity.

Reversal rates and occurrences of mixed percepts were counted during a 5-minute viewing of the ambiguous SFM after the priming experiment. In the instructions, the two possible distinct orientations of the cylinder were described, and mixed percepts when no distinct cylinder with a specific orientation could be distinguished. Participants were instructed to press the F-key when the left side of the cylinder was perceived closest, the K-key when the right side was perceived closest (with the cylinder opening visible), and the space bar when no clear orientation could be seen (mixed percepts).

Finally, questionnaires were administrated to estimate traits such as the separate dimensions of spatial and object imagery (VOSI), the Big-5 personality traits questionnaire including all five dimensions, the autism spectrum quotient (AQ), and the rational and experiential cognitive styles (REI). These were included at the end of the priming and reversals tests.

### Participants

The average age of the 31 (17 female and 14 male) participants was 30 years (Std = 11). They were mainly recruited from the campus and social media, and were given a cinema ticket for compensation.

### Results

Since the number of participants was low for the correlation analyses (as an unexpected consequence of covid-19 restrictions), a two-tailed sensitivity frequentist analyses were performed (G*power 3.1.9.4) after data collection, showing that the required effect size, given 31 participants, α = 0.5, and β = .2, is r = .46, which is a relatively large correlation. The corresponding sensitivity for a two tailed one-sample t-test is d = .52 (a moderate Cohen’s d).

Fig 4B shows that when the test cylinder appeared at a random location around the centrally presented prime cylinder, then all primes influenced the subsequently presented ambiguous cylinder to be perceived with the same orientation as the prime, and by approximately the same percentage, about 70% of trials in all three priming conditions. Table 3 present the results from the one-sample t-tests with population value = 0.5 meaning no influence of the prime, and paired samples t-tests showing that the differences between pairwise comparisons were not statistically significant. The Bayes factors provided support for the absence of any difference .19 < BF_10_ < .22. This means that the results are about five times more likely given no true difference than if there was a true difference, which is considered as moderate evidence for the absence of a difference.

**Table 3:** Influence of primes and differences between primes in Experiment 2.

*Prime conditions*	t(29)	p	BF_10_*	Cohens d
SFM	7.2	< .001	260 000	1.3
Pictorial	7.7	< .001	890 000	1.4
Imagery	6.0	< .001	13 000	1.1
*Differences*	t(30)			
SFM-Pictorial	-.17	.87	.19	-.030
SFM-Imagery	.20	.85	.20	.035
Pictorial-Imagery	.50	.62	.22	.090

The one-sample t-test was used to test the influence of the primes, and the within-sample t-tests for the differences. All tests are two-tailed.

* The Cauchy prior width parameter was set equal to the default value .707.

By comparing Fig 4A and 4B the priming found in Experiment 2 seem to be somewhat reduced compared to the priming from Experiment 1. The difference between the three corresponding priming conditions in Experiment 1 and 2 were tested with independent samples two-tailed frequentist and Bayesian t-tests. The difference between the SFM-prime was statistically significant between Experiment 1 and 2, stronger priming resulted when the prime and test appeared at the same location (t = 3.1, n_1_ = 86, n_2_ = 31, p < .002, d = .66, and BF_10_ = 14 provide strong evidence for a difference). The difference between the pictorial-prime conditions and the difference between the imagery-prime conditions obtained in Experiment 1 and 2 were not significant (t = 1.2, p = .24, BF_10_ = .41, d = .25; and t = .68, p = .50, BF_10_ = .27, d = .14 respectively, where the BF provide evidence against a difference ranging from anecdotal to moderate evidence). Thus, only the SFM priming seems to be reliably location-specific. This provides additional evidence for the involvement of low-level motion-specific processes in the within-cue priming condition (SFM prime followed by the SFM test). All three primes are equally efficient when the test appear at a random position.

The only systematic bias observed by averaging the results across all primes and prime orientations, was that of perceiving the ambiguous SFM cylinder oriented with its left side closest (one sample t-test: t(29) = 3.0, p = .005, BF_10_ = 8.1). No systematic bias to perceive the ambiguous cylinder’s with forward or backward slant was observed. (-.31 < t < .88; .21 < p < .76; .20 < BF_10_ < .40). The left side bias may result from common reading behaviour (scanning from left to right in western societies) initially directing visual attention to the left of any display.

**Fig 5:**
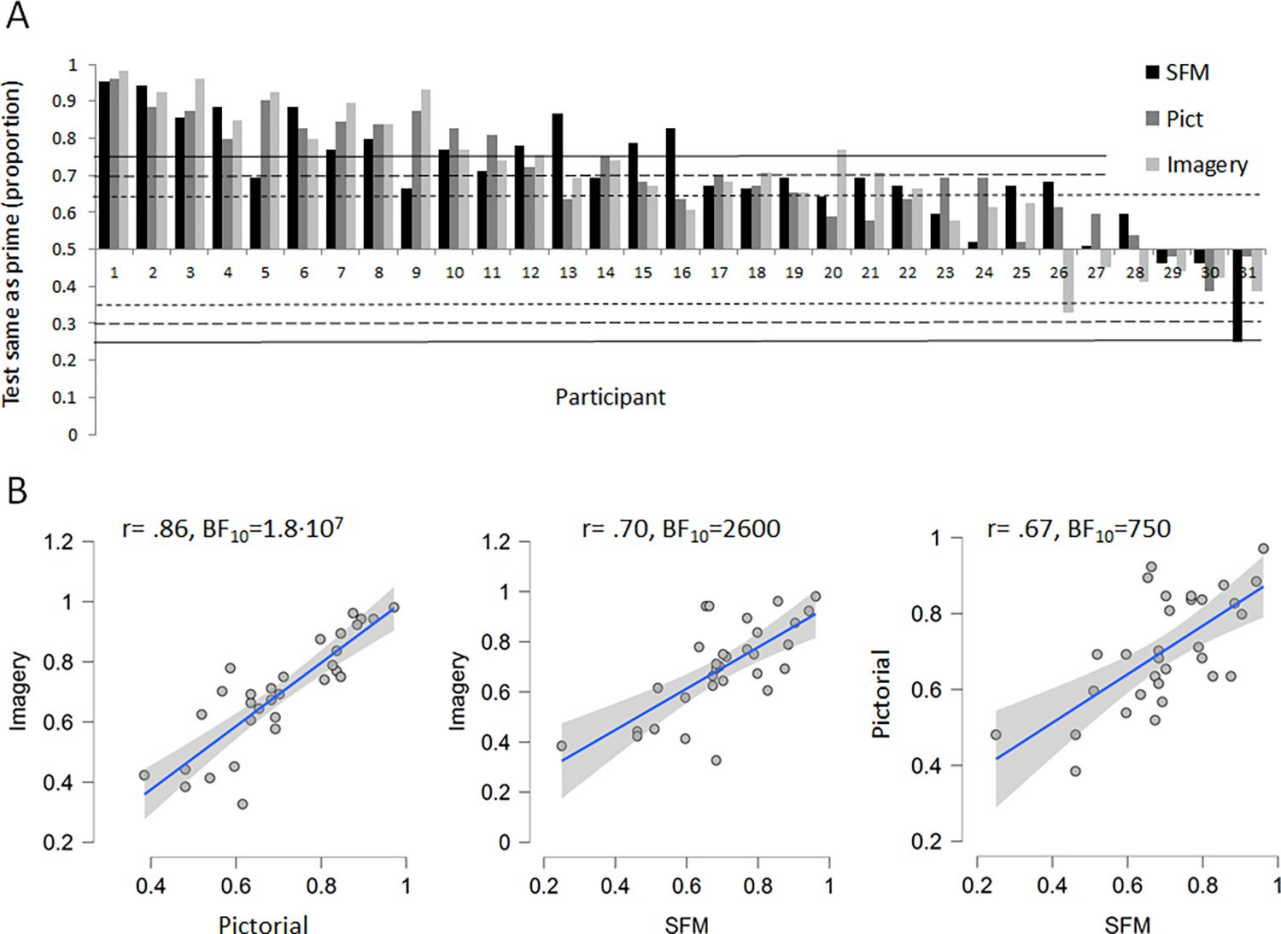
Influence of priming and correlations between prime conditions. (A) The order of participants is arranged from highest to the lowest average prime-effect across the three prime conditions. The bars show the proportion of responses where the test stimulus was perceived with the same orientation as the prime (out of 52 trials, .5 indicates no influence). Horizontal lines: p < .05; dotted line BF > 2; dashed lines BF > 10; solid lines BF > 100. (B) Pearson’s r and Bayes factors are shown in each scatterplot. All p’s < .001, tests are two-tailed. The shaded areas show the 95% CI of the regression lines.

Fig 5A shows the results from each of the 31 participants and can be considered as 31 repeated experiments, which increase the reliability and provide finer grained information than single analyses performed on group averages. The results from each individual and condition are based on 52 responses (also, an option not to respond was permitted when no clear orientation of the test cylinder was perceived, but these occurrences were few and not reported here). Significant deviations from the .5 proportion level, indicating no influence from the prime, is marked with the horizontal dotted, dashed line, and solid lines indicating BF_10_ > 2 (p < .05), BF_10_ > 10, and BF_10_ > 100 respectively. Large individual differences were found, ranging from almost 100% priming to no apparent influence of the prime, with one observer even reporting reliable reversed priming in the SFM condition (BF_10_ ≈ 100), i.e. an adaptation effect.

Fig 5B shows the relations between the influences of primes in the three conditions. The correlation between the imagery priming and pictorial priming was stronger than the correlation between the SFM priming and pictorial priming, and the SFM priming and Imagery priming (z = -2.42, p = .016 and z = -2.02, p = .043 respectively (testing for the equality of two correlation coefficients [73]. Thus, we replicated the result from Experiment 1 with this stimulus setup using a smaller sample. Influences of different primes mediated by processes that share a common origin are likely to correlate to a higher degree than if they are mediated by different processes. So the differences in correlations may imply that even though influences of low-level processes were minimised compared to Experiment 1, the imagery and pictorial priming involve more common top-down processes than the SFM-priming which still involved relatively more low-level processes.

#### Reversals and mixed percepts

With some exceptions [62] mixed percepts are seldom reported in research on ambiguous SFM perception, instead it is often reported that perception switches between just two possible interpretations and the term bistable perception is often used. Here, about 40% of observers reported mixed percepts between 1 and 10 times while viewing the ambiguous SFM cylinder during 5 minutes. The number of reported and total time of mixed percepts were highly correlated (Kendall’s Tau-b = .95, p < .001, BF_10_ = 5.8∙10^10^). Hence, in the following analyses involving mixed percepts we use the number of reported mixed percepts. The average number of reversals during the 5 minutes of observation was 9.8, ranging between 0 (seven observers) and 83 (one observer). Table 4 shows that the reported number of reversals and the number of mixed perceptions were highly correlated. No reliable correlations were found between the influences of priming and reversal rates or between influences of priming and mixed perceptions, the Bayes factors inform that these correlations provide weak support for true correlations, or provide weak to moderate support for there being no true correlation.

**Table 4:** Correlations Tau-b, between priming (SFM, pictorial, and imagery), reversals, and mixed percepts.

	SFM	Pictorial	Imagery	Reversals
Reversals	.23†	.24†	.29*	
N-mixed	.06†	.16†	.15†	.56***

Shown are the Kendall’s Tau-b correlations between influences of the primes and the number of reversals and mixed percepts. Although the distributions of reversals and mixed percepts were skewed Pearson correlations were similar to Kendall’s Tau-b.

† .26 < BF_10_ <. 1.4.

*p < .05 (BF_10_ = 3).

*** p < .001 (BF_10_ = 3183).

#### Relations between ambiguous SFM perception and personality traits

**Fig 6:**
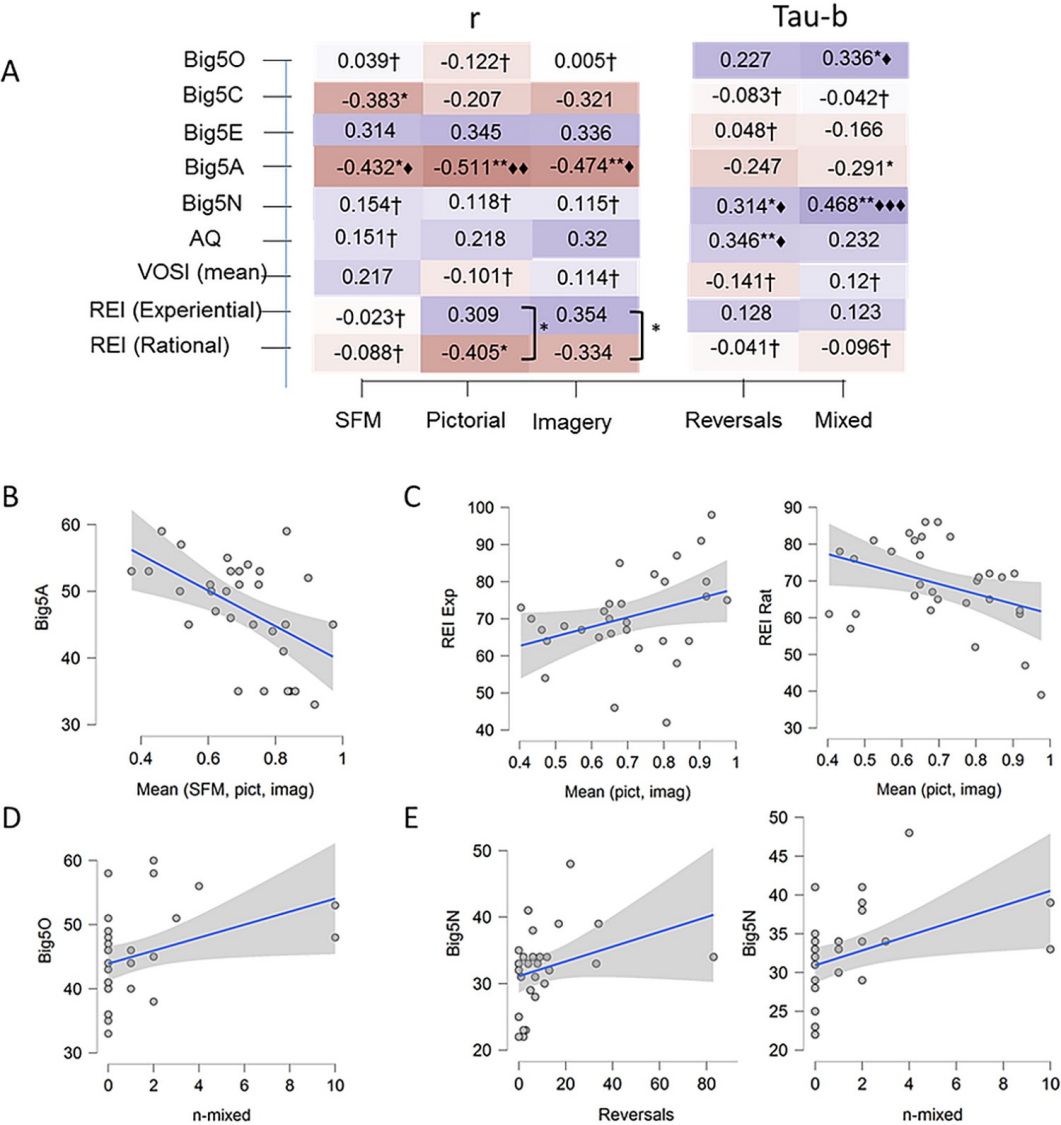
Heat-map and scatterplots. A. Correlations are shown between the influences of priming (SFM, still image, and imagery) and the personality traits (Pearson’s r), and number of reversals and mixed perceptions and personality traits (Kendall’s Tau-b). Tests are two tailed: * p < .05, ** p < .01, ♦BF_10_ > 3, ♦♦BF_10_>10, ♦♦♦BF_10_ > 100 (in support for a correlation). The sign † indicates cases where the result is 3 to 5 times more likely due to an absence of a correlation than if there was a correlation in the population. The staples between the experiential and rational cognitive styles indicate significant differences between correlations. B. Scatterplot between agreeableness scores and priming averaged across all three prime conditions. C. The scatterplots between the experiential and rational cognitive modes as scored by REI and the influence of the SFM-prime and the imagery-prime presented separately. D. The scatterplots between openness scores and the number of mixed percepts. E. Separate scatterplots are shown between reversals and neuroticism, and mixed percepts and neuroticism.

Fig 6A shows a heat map displaying the correlations between perception of the ambiguous SFM stimulus (influence of priming from the three priming conditions, reversals, and duration of mixed percepts) and psychological traits as scored by the questionnaires (Big-5, AQ, VOSI, and REI scores). Neither the object nor spatial imagery scores from the VOSI questionnaire were correlated to priming, reversals or mixed percepts. Therefore, the object and spatial imagery scores were averaged. Since the sample is relatively small, the distribution of the number of reversals and mixed percepts were skewed, and contain ties, the non-parametric Kendall’s Tau-b (adjusted for ties) are presented for the reversals and mixed percepts relations with the psychological traits. Fig 6B–6E shows a selection of the most interesting significant scatterplots. Fig 6B show the scatterplot between all three primes averaged and the agreeableness dimension of the Big-5 personality test (for the SFM-prime r = -.43; for the pictorial-prime r = -.51; and for the imagery-prime r = -.47). Fig 6C show the scatterplot between the experiential and rational cognitive styles as scored by REI, and the influence of the pictorial-prime and the imagery-prime averaged. No correlations between REI scores and the influence from the SFM-prime were found in our data. Fig 6D show the scatterplot between the Big-5 dimension openness and number of mixed percepts. Fig 6E show the scatterplots between reversals and the neuroticism dimension of Big-5, and the number of mixed percepts and neuroticism scores.

Although a positive correlation between VOSI scores and influence of priming was predicted, no such correlation was found for any priming condition. The BF’s provide moderate evidence for an absence of correlations for priming-VOSI scores, priming-openness, and priming-neuroticism. The BF’s were uninformative about the correlations between priming-extraversion, and priming-conscientiousness, and no expectations or hypotheses concerning these traits were formulated. Further, no evidence for any correlation between priming and AQ-scores was obtained. An interesting finding, not expected, was the moderate to strong evidence for a negative correlation between priming and scores on agreeableness, as indicated by the BF’s.

Whereas the correlations between influences from both the pictorial and imagery primes and cognitive styles (scored by REI) were in predicted directions (positive for the experiential and negative for the rational style), no reliable conclusion can be made from this small sample. The difference between these correlations, however, were statistically significant (for the still image prime p = .025 and for the imagery prime p = .018 respectively using a two-tailed test [73], no BF calculator was available for this inference test). The Bayesfactor provided support for an absence of a correlation between both cognitive styles and SFM-priming which was influenced by low level SFM-specific processes as suggested by the previous analyses. Although the relations between priming and cognitive styles are admittedly weak, they inspire to further investigations.

Some interesting relations between reversals, mixed percepts, and personality traits were found. The results from Antinori et al. [49] showing a positive correlation between the Big-5 trait openness and occurrences of mixed percepts while observing a binocular rivalry stimulus was also observed with our ambiguous SFM stimulus (BF_10_ = 6.9). No correlations were obtained between openness and frequency of reversals. This mimic the results from Antorini et al. who found no correlation between switching rates in binocular rivalry for perceiving either the left or right image, and openness. The trait neuroticism was positively correlated with the number of reversals (BF_10_ = 4.5), and highly so to the number of mixed percepts (BF_10_ = 166). As hypothesised, this finding is probably related to previous findings of an elevated frequency of switching while viewing binocular rivalry images for anxious compared to non-anxious individuals [46].

No correlations were found between reversals or mixed percepts and conscientiousness, extraversion, agreeableness, imagery or cognitive styles. The BF’s indicate moderate evidence for an absence of any correlation or is uninformative.

Contrary to previous reports of reduced reversal rates while viewing the Necker cube [61], and rotation direction in SFM [62] for those clinically diagnosed with autism, we found a positive correlation between AQ scores for our non-clinical sample and the number of reported reversals of our ambiguous orientation SFM stimulus (BF_10_ = 8.5). No reliable correlations between AQ scores and reported mixed percepts or influences of the primes were found.

Correlation table containing all correlations between variables from the questionnaires is provided as supporting information (S1 Table), and the data set behind the analyses from Experiment 2 result section is provided as supporting information (S1 Data, in sheet 2).

## Discussion

### Priming of ambiguous orientation in SFM

In two Experiments, we found that priming of an SFM test stimulus with an ambiguous 3-D orientation occurred in all prime conditions, SFM prime, pictorial prime, and imagery prime, followed by the SFM test. The SFM prime was more efficient when the SFM test appeared at the same position as the prime than at a random position, no such influence of position was found for in the other prime conditions. Comparison between the results obtained in Experiment 1 and 2 showed that pictorial priming occurred irrespectively whether the test appeared at the same position or a random position around the prime (i.e. position invariance). The imagery prime was also position invariant. In both experiments, the correlation between the influence from the pictorial and imagery primes were stronger than the correlation between the SFM-prime and pictorial-prime, and SFM-prime and imagery prime.

Ambiguous SFM rotation direction is known to be influenced by motion specific priming [24,25,27], and our results provide some evidence that our SFM-priming of orientation also involve some residual lower level processes. Cue invariant priming, position invariant priming, and particularly the imagery-priming, likely result from top-down activity that may originate from the frontal lobes that modulates neural responses in temporal cortical regions such as the superior temporal sulcus (STS) known to be involved in the perception of 3D shape [74] irrespectively of the cue defining the shape [75]. Thus, our results suggest the involvement of a cue invariant hypotheses generator at a high level used to predict sensory input. Such cue invariance has also been reported in cross-cue adaptation to surface slant [76], and simultaneous slant contrast [77].

A confound in the priming in Experiment 1 is the influence of fixation. The fixated end of the cylinder was reported as closest on most trials in the fixation condition in Experiment 1 which mimics the results obtained with the Necker cube [8]. A possible explanation to the influence of fixation is that an association between fixation and relative distances to surfaces might arise naturally since objects are typically opaque and only the surfaces facing the viewer are visible from the viewer’s perspective. If participants systematically used the closest part of the prime as a cue to direct attention to the same end of the test stimulus when it appeared (voluntarily or involuntarily against instructions), there would be little difference between the fixation condition and the prime conditions. However, we found weak evidence for this. The correlations between the influence from the fixation-condition, where participants were instructed to fixate either end of the test cylinder, and the other prime-conditions were much lower than correlations between the prime-conditions (at most 10% shared variance compared to between 35 to 55% shared variance). In Experiment 2, besides targeting position-invariance in priming, the random position of the test-cylinder along a circular path centred at the prime cylinder also minimised the influence of the prime as a potential signal for directing attention. Another possible confound in priming could be inter-trial influences. There is a possibility of correlations between trials in the priming experiment, but since the sign of orientation of the prime was randomised for each trial, such inter-trial influences would counteract the influence of the primes presented in each trial. In addition, the possible influences of previous trial are likely to be masked by the prime preceding the test.

### Reversals and mixed percepts in ambiguous SFM orientation

In Experiment 2, in addition to the priming experiment, the perceived reversals and mixed percepts were counted during 5 minutes of observation of the ambiguous SFM stimulus used as a test-stimulus in the priming experiment. Mixed perception has previously been used as a response option when measuring perceptual switches of rotation direction in an ambiguously revolving SFM stimulus, but occurrences of mixed perceptions in that study were rare during the 90 sec observation used [62]. We found that the frequency of reversals and the frequency of mixed percepts were positively correlated. Interestingly, we found no correlation between the influence of priming and the number of reversals, or the influence of priming and the number of mixed percepts, so these tasks seem to target different processes. Spontaneous perceived reversals from ambiguous stimuli are thought to involve competing neural populations cycling through adaptation and recovery. Reversals may also involve both a bottom-up flow of prediction errors resulting from evidence for the suppressed percept, and top-down flow from the hypothesis generator that attempt to explain the sensory signals, as described in the predictive processing (PP) framework [15,78]. In line with PP, the face-vase and the Necker cube stimuli elevates both top-down and bottom-up activity compared to stable versions of these figures [40].

### Relations between priming, reversals, mixed percepts, and personality traits

What individual characteristics, or traits, may relate to the large variability in the influences of the primes, reversal rates and mixed percepts between individuals? Although we expected a correlation between imagery questionnaire scores (VVIQ in Experiment 1 and VOSI in Experiment 2) and the influence of priming, especially the imagery priming, no such correlation was found. The imagery scores from both VVIQ and VOSI were diverse and normally distributed, so a restriction of range cannot explain this lack of correlation. VVIQ scores ranged between 17–77 (max possible spread was 16–80), with mean 52 and std 13.4, and the VOSI ranged between 26–58 (max possible spread was 14–70), with mean 46 and std 7.9. The Bayes factors instead suggested that the data were more likely given a lack of correlation than if there was a correlation in the population. The vividness experienced during imagery has been associated with higher activation of top-down connectivity [79]. When measured as a stable trait it is related to increased overlap between perception and imagery throughout the entire cortical hierarchy [80]. Imagery, however, is not just a weak form of perception. The distribution of object information across visual areas is strikingly different during imagery and perception [81], and visual priming may be related more to perceptual processes than visual imagery. In addition, imagery priming might have resulted in a high-level conceptual priming rather than visual priming. In such case, the line having a circle at one of its end might have become associated with the semantic meaning of the verbal instructions, resulting in an efficient conceptual prime rather than a visual one.

A surprising result in relation to the Big-5 inventory was the large negative correlations found between the trait agreeableness and influence of priming across all prime three prime conditions (-.43 < r < -.51). The three correlations were 3.7, 7.1 and 13.7 times more likely obtained given a negative correlation than if there was no correlation in the population. The origin of this negative correlation is unclear. People scoring high on agreeableness tend to display a higher degree of prosocial behaviours. They are also more inclined to be conscious of and accommodating to other people’s wants and needs; therefore, the negative correlation here provides evidence against the possibility that demand characteristics might have influenced our results. Instead, the more agreeableness an individual has scored on the Big- test, the less likely is this individual to be influenced by the prime. In addition, the two possible perceived orientations of the ambiguous cylinder during the two seconds of presentation in the end of each trial were distinct and participants found the task easy to comprehend, so observers were likely reluctant to respond contrary to their perceptions of the test cylinder. Demand characteristics are likely to have more impact when response options are not clearly discriminable. That the SFM priming was less correlated to the pictorial priming and imagery priming than the latter two were related is difficult to explain by assuming priming effects due to common demand characteristics. It is also very unlikely that responses were mistakenly made to the prime stimulus. The response options (key presses) were not available until the test stimulus had been presented and the prime and test were well separated in time (2 sec), and the instructions clearly pointed out that the response should indicate the perceived orientation of the finally presented cylinder in each trial.

In Experiment 1 we hypothesised that the Big-5 openness dimension, where one of the facets is imagination, could potentially be linked to top down driven priming. No such relation was found. In Experiment 2, we included all five dimensions of the Big-5, measured the number of reversals, and mixed percepts. Our sample in Experiment 2 was quite small for the analysis of correlations with personality variables and larger samples should be used in attempts of replications to increase external validity. However, the non-parametric Kendall’s Tau-b, which is used here, is conservative and robust when samples are small, include outliers and ties. Both the number of reversals and mixed percepts was measured during the session of five minutes of observation of the ambiguous SFM stimulus, inspired by the findings that openness and mixed percepts during binocular rivalry seem to be linked [49]. The correlation (Kendall’s Tau-b = .336) obtained between the occurrences of mixed percepts and the Big-5 trait openness was 6.8 times more likely given a true correlation than if there was no correlation in the population as implied by the Bayes factor. The correlation between reversal rates and openness was not statistically significant, the Bayes factors were between 1/3 and 3 and thus unable to inform us whether the data was more likely given a true correlation or an absence of a correlation in the population. Both these results replicate the Antinori et al results [49,53] who used a binocular rivalry stimulus, but we obtained larger effect size in the correlation between mixed percepts and openness suggesting that our ambiguous SFM stimulus is better to target the common processes. The correlation between openness and mixed percepts may be explained through the predictive processing concept of granularity, referring to level of detail in predicting sensory information, introduced by Kwisthout and Rooij [82]. Parallels can be drawn to divergent thinking, such as coming up with multiple uses for one object, found in persons high in openness. That we could replicate the previous results obtained by binocular rivalry stimuli with our ambiguous SFM stimulus is interesting due to the difference between binocular rivalry and other forms of ambiguous figures (in rivalry the visual input from one eye must be entirely suppressed).

Also as hypothesised, we found a positive correlation between the Big-5 trait neuroticism and reversal rates. Neuroticism and anxiousness are linked, and previous results show that the degree of anxiousness is correlated with reversal rates while viewing a binocular rivalry stimulus [46,47]. An additional finding was that the occurrences of mixed percepts and neuroticism were strongly related, whereas priming was not related to neuroticism.

Previous studies have reported that clinically diagnosed autistic people report no difference in switching rates in binocular rivalry [60], or lower frequency when observing binocular rivalry stimuli [57–59] and Necker cubes [61]. We found, instead, a positive correlation between AQ scores and number of reversals of the ambiguous SFM stimulus in our non-clinical sample. Besides possible sample noise, the difference between studies might result from the different samples investigated, clinical vs. non-clinical, or that the difference comes from using different stimuli. For example, different stimuli may target top-down and bottom-up processes to varying degrees. In line with previous results [83] we found that scores on the AQ enquiry and scores on the Big 5 trait neuroticism were positively correlated, whereas the trait agreeableness and AQ scores were negatively correlated (see S1 Table). This cross validates these trait measures and is consistent with the pattern of relations found between reversals and these traits.

We predicted that scores on experiential and rational cognitive mode might be related to influences from top-down driven priming. The correlations provide hints that individuals scoring high on the experiential cognitive mode may be more influenced by top-down driven priming (particularly the pictorial and imagery primes), whereas the opposite holds for individuals scoring high on the rational mode. The differences, between these correlations were statistically significant for the still-image prime and imagery prime condition, which, according to our results from the priming, activate top-down processes more than the SFM prime. Replications with larger sample to increase external validity in future studies are needed to provide a clearer picture of the relation between individual variability in cognitive modes and perception in the form of influences of high-level priming.

### Final remarks

Different types of ambiguous stimuli may target different processes, and different methods such as counting reversals, counting mixed percepts, priming, or adaptation may also target different processes, which have to be considered in research on ambiguous stimuli in general. Our research, not only contribute to the specific knowledge concerning top-down influences in SFM-perception but also addresses questions concerning the relation between different psychological processes. Research in perception, personality and cognition, with few exceptions, are typically performed as separate research fields without fruitful crosstalk that could aid theory development. Thus, the results presented here add important clues, not only about mechanisms behind ambiguous SFM-perception per se, but also add evidence to bridge this gap between research fields.

## Supporting information

S1 Movie Demo of the ambiguous SFM test stimulus. (MP4)

S2 Movie Demo of the disambiguated SFM prime stimulus. (MP4)

S1 Table Correlation table for all correlations between variables from the questionnaires. (PPTX)

S1 Data Sheet 1: Experiment 1; sheet 2: Experiment 2. (XLSX)
