# Supplementary material for: Priming and reversals of the perceived ambiguous orientation of a structure-from-motion shape and relation to personality traits
Source: PLoS One. 2022 Aug 26;17(8):e0273772. doi: 10.1371/journal.pone.0273772 (PMC9417019; doi:10.1371/journal.pone.0273772)
Supplement: S1 Table — (PPTX) [file pone.0273772.s003.pptx]

## Slide 1
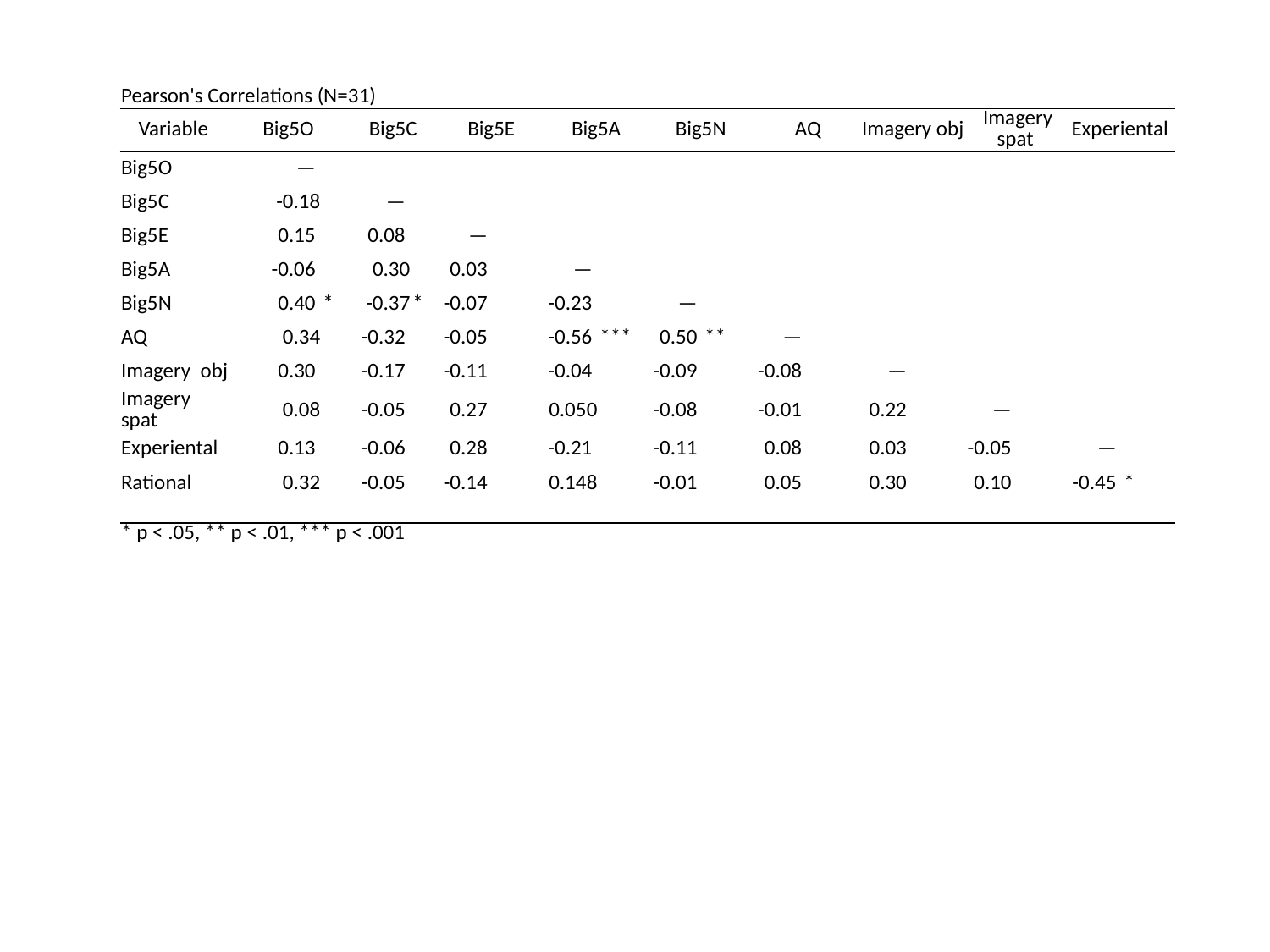

| Pearson's Correlations (N=31) | | | | | | | | | | | | | | | | | | |
| --- | --- | --- | --- | --- | --- | --- | --- | --- | --- | --- | --- | --- | --- | --- | --- | --- | --- | --- |
| Variable | Big5O | | Big5C | | Big5E | | Big5A | | Big5N | | AQ | | Imagery obj | | Imagery spat | | Experiental | |
| Big5O | — | | | | | | | | | | | | | | | | | |
| Big5C | -0.18 | | — | | | | | | | | | | | | | | | |
| Big5E | 0.15 | | 0.08 | | — | | | | | | | | | | | | | |
| Big5A | -0.06 | | 0.30 | | 0.03 | | — | | | | | | | | | | | |
| Big5N | 0.40 | \* | -0.37 | \* | -0.07 | | -0.23 | | — | | | | | | | | | |
| AQ | 0.34 | | -0.32 | | -0.05 | | -0.56 | \*\*\* | 0.50 | \*\* | — | | | | | | | |
| Imagery obj | 0.30 | | -0.17 | | -0.11 | | -0.04 | | -0.09 | | -0.08 | | — | | | | | |
| Imagery spat | 0.08 | | -0.05 | | 0.27 | | 0.050 | | -0.08 | | -0.01 | | 0.22 | | — | | | |
| Experiental | 0.13 | | -0.06 | | 0.28 | | -0.21 | | -0.11 | | 0.08 | | 0.03 | | -0.05 | | — | |
| Rational | 0.32 | | -0.05 | | -0.14 | | 0.148 | | -0.01 | | 0.05 | | 0.30 | | 0.10 | | -0.45 | \* |
| | | | | | | | | | | | | | | | | | | |
| \* p < .05, \*\* p < .01, \*\*\* p < .001 | | | | | | | | | | | | | | | | | | |
